# Clonal hematopoiesis of indeterminate potential and its impact on patient trajectories after stem cell transplantation

**DOI:** 10.1371/journal.pcbi.1006913

**Published:** 2019-04-26

**Authors:** Derek S. Park, Afua A. Akuffo, David E. Muench, H. Leighton Grimes, Pearlie K. Epling-Burnette, Philip K. Maini, Alexander R. A. Anderson, Michael B. Bonsall

**Affiliations:** 1 Mathematical Ecology Research Group, Department of Zoology, University of Oxford, Oxford, United Kingdom; 2 Department of Integrated Mathematical Oncology, H. Lee Moffitt Cancer Center, Tampa, Florida, USA; 3 Department of Immunology, H. Lee Moffitt Cancer Center, Tampa, Florida, USA; 4 Division of Immunobiology and Center for Systems Immunology, Cincinnati Children’s Hospital Medical Center, Cincinnati, Ohio, USA; 5 Wolfson Centre for Mathematical Biology, Mathematical Institute, University of Oxford, Oxford, United Kingdom; University of California Irvine, UNITED STATES

## Abstract

Clonal hematopoiesis of indeterminate potential (CHIP) is a recently identified process where older patients accumulate distinct subclones defined by recurring somatic mutations in hematopoietic stem cells. CHIP’s implications for stem cell transplantation have been harder to identify due to the high degree of mutational heterogeneity that is present within the genetically distinct subclones. In order to gain a better understanding of CHIP and the impact of clonal dynamics on transplantation outcomes, we created a mathematical model of clonal competition dynamics. Our analyses highlight the importance of understanding competition intensity between healthy and mutant clones. Importantly, we highlight the risk that CHIP poses in leading to dominance of precancerous mutant clones and the risk of donor derived leukemia. Furthermore, we estimate the degree of competition intensity and bone marrow niche decline in mice during aging by using our modeling framework. Together, our work highlights the importance of better characterizing the ecological and clonal composition in hematopoietic donor populations at the time of stem cell transplantation.

## Author summary

We investigated the impact of clonal hematopoiesis of indeterminate potential on stem cell transplantation outcomes. Interclonal competition intensity was identified as an important determinant of ultimate hematopoietic trajectory. We also investigated the impact of age-related marrow changes and show that, from a mouse model, these ecological parameters can be estimated.

## Introduction

Hematopoiesis is a clonal process [1]. Hematopoietic stem cells (HSCs) are a small population of multipotent cells with self-renewal capacity for lifelong hematopoiesis. However, the very self-renewal capabilities of HSCs which make them so unique and versatile also make them vulnerable to the accumulation of somatic mutations [2]. Recently, studies have demonstrated that somatic mutation accumulation in hematopoietic cells in key cancer-related genes is cumulative over a patient’s lifetime [3–5]. The acquisition of somatic mutations that drive clonal expansion of hematopoietic stem cells is considered hematopoiesis of indeterminate potential (CHIP) [1].

From an evolutionary and ecological perspective, CHIP can be understood as the slow process of clonal diversification in the specific ecosystem of the bone marrow. Taking place over the course of a patient’s lifetime, the stem cell dynamics of hematopoiesis involve genetic variation, inheritance and potentially selection pressures. These are the pre-requisites necessary for evolution to occur and can eventually lead to mutations that convey dominant phenotypic changes and clinically manifest as malignancies [6].

Importantly, CHIP underscores the uncertainty in terms of health outcomes for patients that have these mutant hematopoietic clones. While CHIP has been linked with both cancers and other diseases [7], the challenge of the condition is that the heterogeneity and range of genetic variation that could be present in a patient are poorly characterized and do not guarantee disease progression [1].

An area where the impact of CHIP has been less studied is the interaction between clonal diversity and stem cell transplantation. Stem cell or bone marrow transplantation (BMT) remains one of the most common treatments for myeloid and lymphoid leukemias. Transplantation types include allogenic donor marrow or the patient’s own marrow harvested before administration of high dose chemotherapy (autologous) [8].

However, the process of autologous BMT raises potential causes for concern when examined from an ecological point of view. Specifically, autologous BMT involves the sub-sampling of a genetically heterogeneous population and then repopulation in a recently emptied bone marrow microenvironment. It applies a population bottleneck to multiple clones without any clinical assessment of the composition of the reintroduced clones. Additionally, many of the cancers which are treated with autologous BMT occur in elderly patients [9]. Nationally, in the United States between 2012 and 2016, roughly 70% of all autologous BMT patients were older than 51 years of age with approximately 40% of all patients being older than 61 years [10].

The age-association with CHIP has been examined to show that elderly patients at 65 years and above are far more likely to have both driver and non-driver mutations in their hematopoietic cells [3, 4, 11]. These include mutations in important cancer-protective genes such as p53 [3]. The frequency and potential impact of these clones in BMT remains unexamined. Indeed, clinical assessments prior to autologous transplantation do not consider clonal composition [12], which raises the troubling possibility that autologous BMT from donors with CHIP may lead to the development of post-transplantation acute leukemias derived from a preexisting clonal population. Moreover, allogenic transplantation outcomes may be similarly impacted by the age of the donor.

In order to investigate how clonal composition impacts transplantation outcomes, we use a mathematical framework that integrates the competition dynamics between healthy and mutant clones with the unique biological feedback mechanisms of the hematopoietic system. We specifically investigate the repopulation trajectories of patient bone marrow after differing compositions of healthy and mutant cells were transplanted. Of main concern is how the intensity of competition and the disparity in growth rate between healthy and mutant clones impact overall bone marrow outcomes.

Finally, we broaden our investigation to understand the possible interactions of CHIP and patient marrow conditions. Just as aging leads to CHIP in donors, older patients also have compromised bone marrow. Specifically, aging has been associated with a decline in the capacity of bone marrow niches to support stem cells [13]. We investigate how differing degrees of bone marrow niche decline might interact with CHIP to influence transplantation outcomes. In this process, we use comparative stem cell measurements from mouse cohorts to estimate clonal competition parameters. In doing so, we suggest that poor patient marrow condition can potentially magnify the detrimental outcomes associated with CHIP.

Our analyses underscore the importance of understanding the clonal composition of cells that may be undergoing transplantation and also the bone marrow microenvironment into which they are transplanted. The level of clonal competition has the potential to lead to very divergent outcomes in final patient hematopoietic states. However, ecological modeling provides a potential avenue for estimating these parameters and understanding the diversity contained within CHIP.

## Materials and methods

### Model design

**Fig 1:**
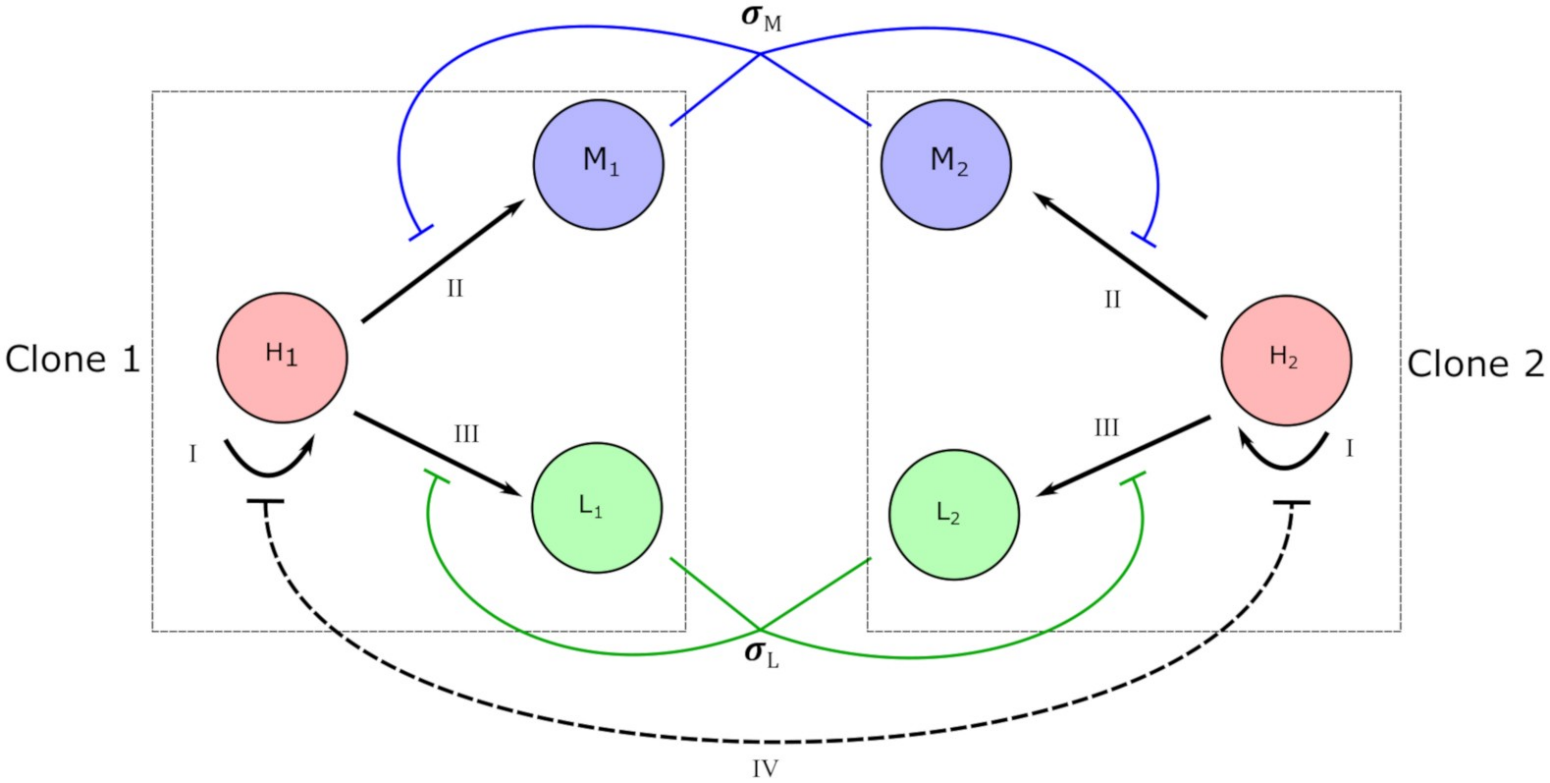
Model interactions in the hematopoietic system. The model system consists of two competitive hematopoietic clones. Clone 1 is designated as healthy cells while clone 2 is designated as a pre-malignant but mutant lineage. Both clones are controlled by shared, global feedback signals. Within each clone there is hematopoietic stem cell (HSC) renewal (cell compartments *H*_1_ and *H*_2_, arrow I), delayed differentiation to myeloid cells (cell compartments *M*_1_ and *M*_2_, arrow II), and delayed differentiation to lymphoid cells (cell compartments *L*_1_ and *L*_2_, arrow III). The delay is represented by term *τ* in the equations. Both myeloid and lymphoid cells exhibit negative feedback control (*σ*_*M*_ and *σ*_*L*_, respectively) as they approach homeostatic levels. Simultaneously, there is Lotka-Volterra competition in the renewal of HSCs representing competition for a common pool of cytokines (line IV).

In order to model clonal competition during hematopoiesis, the bone marrow system was divided into healthy and mutant, pre-malignant clones. In addition to hematopoietic stem cells (HSCs), mature myeloid, and mature lymphoid cells (Fig 1) were also modeled. HSCs differentiate into mature cells after a period of time (*τ*) that represents cell cycle times as well as transit times from stem to progenitor then finally differentiated states.

Recent studies with greater genetic resolution have demonstrated that even in healthy patients, the hematopoietic population is not uniform [3]. As patients age, there is increasing genetic heterogeneity that leads to a divergence between healthy and mutant hematopoietic clones [1]. Even if these mutant clones are not clinically defined as cancerous, they often exhibit mutations that could potentially lead to cancer later on [1]. In addition, these mutants are known to be different than healthy populations in multiple ways including their proliferation rates and their consumption of resources in the bone marrow microenvironment [1].

In this context, the goal of our modeling framework was to create a model of hematopoietic competition which takes into account the unique physiological feedback channels which control hematopoiesis [14]. We have modeled two separate but competing clonal lineages to represent the broad qualitative division of HSCs into healthy and mutant clones which were designated as clones 1 and 2, respectively. However, we have made their differences quantitative to characterize, more clearly, how the degree of differences in parameters, such as competition intensity and growth rates, can impact reconstitution and disease dynamics. The degree of divergence in the healthy and mutant lineages is reflected in these ecological parameters. By representing the hematopoietic stem cell system as a composition of two major clonal lineages with different ecological characteristics, we both reflect current understanding of the diversity of HSCs in CHIP, but also retain and allow enough flexibility in the model framework to examine the spectrum of differences between these two extremes. We are able to quantitatively interrogate the relationship between these healthy and mutant lineages in overall HSC dynamics.

Other mathematical models, notably from Ashcroft et al. [15], have looked at clonal dynamics in transplantation from a stochastic perspective. However, while those analyses approximated clonal competition and growth as a Moran process, they do not take into account some of the biological complexities that distinguish the hematopoietic system. Our framework seeks to extend these studies by explicitly describing physiological demand as being a driver of differentiation [16]. In addition, we constrain our analysis to dynamics in the bone marrow, but incorporate daughter lineages of mature lymphoid and myeloid cells, as well as HSCs.

### Hematopoietic stem cell dynamics

$$\begin{array}{c} \frac{dH_{1}}{dt}=\underset{I}{\underset{︸}{r_{1}H_{1}(1-\frac{H_{1}+h_{2}H_{2}}{K_{H}})}}-\underset{II}{\underset{︸}{\gamma H_{1}\phi \sigma_{M}-\gamma H_{1}\left(1-\phi \right)\sigma_{L}}}-\underset{III}{\underset{︸}{\delta_{H}H_{1}}} \end{array}$$ (1)

$$\begin{array}{c} \frac{dH_{2}}{dt}=r_{2}H_{2}(1-\frac{H_{2}+h_{1}H_{1}}{K_{H}})-\gamma H_{2}\phi \sigma_{M}-\gamma H_{2}\left(1-\phi \right)\sigma_{L}-\delta_{H}H_{2} \end{array}$$ (2)

$$\begin{array}{c} \sigma_{M}=exp(-\frac{M_{1}+M_{2}}{K_{M}}),\sigma_{L}=exp(-\frac{L_{1}+L_{2}}{K_{L}}) \end{array}$$ (3)

Our framework models healthy (*H*_1_) and mutant (*H*_2_) hematopoietic stem cells, coupled to the dynamics of myeloid (*M*_1_, *M*_2_) and lymphoid (*L*_1_, *L*_2_) cells (see Fig 1). The two clones that are modeled interact with each other through interspecific (Lotka-Volterra) competition dynamics (*h*_1_, *h*_2_) in expression *I* of Eqs 1 and 2. There is a common carrying capacity of HSCs (*K*_*H*_) which is limited by the availability of cytokines and other cellular growth factors. These interspecific competition dynamics are contained in a general logistic growth model with the maximum growth rate of stem cells described by *r*_1_ and *r*_2_. While it is true that hematopoietic stem cells have been further classified depending on subsets of cell surface markers associated with differences in proliferative behavior, we have chosen to model them as one compartment with the dynamics focusing instead on the competition between the clones.

Competition dynamics between healthy and leukemic stem cells have been explored in multiple other modeling paradigms. Our model seeks to describe pre-leukemic clones involved in CHIP instead of explicit competition between HSCs and leukemic stem cells. Other modeling works such as [17] have approached hematopoetic competition dynamics with an explicit division between healthy and malignant stem cells. In our framework, however, by implementing competition coefficients, we can describe dynamics in which the niches of different clones overlap and increase competition. This is in contrast to other systems which have used fixed niches and competition intensities [17]. Finally, in our daughter cell populations (see Eqs 4 and 5 below), we implement a delay (*τ*), which we show is significant in the context of recovering healthy cell levels post-transplantation. These previous competition models, even if using a similar differential equation method as ours, do not take developmental delays into account.

A principal source of stem cell loss comes from differentiation to, eventually, mature myeloid and lymphoid cells and is modeled in expression *II*. Differentiation loss is due to a series of factors. Firstly, differentiated cells do not equally become myeloid or lymphoid cells. Instead, there is a specific fraction of cells which differentiate into the myeloid lineage (*ϕ*) with the remainder becoming lymphoid cells. Lineage determination fractions are then moderated by lineage-specific physiological demand [14, 16]. Eq 3 represents the demand terms (*σ*_*M*_, *σ*_*L*_) which reflect how differentiation can increase for each lineage based on physiological necessity. Importantly, this is one of two mechanisms by which the two clones are intertwined (with the other being the interspecific stem cell competition dynamics).

Our model utilizes a demand-control design that integrates the current levels of myeloid and lymphoid cells and ties them to physiological demand [18]. The demand terms reflect the biological reality that stem cells are under constant feedback control. Both local (adhesion, niche-specific growth factors) and remote (hormones such as erythropoietin) mechanisms act to either promote or suppress stem cell activity based on the overall state of the hematopoietic system and the body. As in [18], feedback mechanisms are dependent on the fully differentiated cells (in this case the myeloid and lymphoid cells). Previous models [19] have used the means of the two clones’ output in calculating feedback. We have a similar implementation that takes into account the output of both clones but instead evaluates them relative to what is necessary to satisfy physiological demand. Dynamically, this demand signal decreases as the total myeloid and lymphoid cells reach the biologically-satisfying carrying capacities (*K*_*M*_, *K*_*L*_). The gamma term (*γ*) represents a scaling relationship between physiological demand and differentiation fraction for the HSC compartment. Used together, our model views homeostasis as a state that is achieved when the final, mature myeloid and lymphoid daughter cells are close to carrying capacity. As cell populations drop from that desired level, there is an increase in upstream stem cell differentiation demand in order to remedy this gap.

Finally, stem cells undergo apoptosis via expression *III*. While healthy stem cell apoptosis rates are relatively low due to their infrequent cell cycling, aging-related bone marrow degradation can lead to elevation of stem cell death (see below). It should be noted, though, that this is an approximation that our model makes over the short term since our primary investigation is to simulate repopulation. Over the course of a lifetime, there are mutation accumulation processes which lead to the emergence of CHIP [3]. However, there are also aging processes which change the growth and division rates of stem cells. Other models have sought to quantitatively characterize this stem cell aging dynamic by dividing stem cell populations into dormant and proliferative stages [20]. In the short post-transplantation time frame, however, we make the approximation that these growth and death rates are invariant.

### Mature myeloid and lymphoid dynamics

$$\begin{array}{c} \frac{dM_{i}}{dt}=\underset{I}{\underset{︸}{\alpha_{M}\phi H_{i}\left(t-\tau \right)}}-\underset{II}{\underset{︸}{\delta_{M}M_{i}}} \end{array}$$ (4)

$$\begin{array}{c} \frac{dL_{i}}{dt}=\alpha_{L}\left(1-\phi \right)H_{i}\left(t-\tau \right)-\delta_{L}L_{i} \end{array}$$ (5)

Myeloid and lymphoid cells mature after a specific delay, *τ*, in expression *I*. A common delay term was used for both myeloid and lymphoid cells due to the high overlap in the variability of maturation times for lymphoid and myeloid cells. An alternative implementation of delay could have been a distributed delay. The common formulation is to use a gamma distribution as a representation of cells which are transiting through a series of compartments (the ‘linear chain’ as in [21]). However a fundamental assumption of the linear chain model and distributed delays is that the flux (number of cells transiting through each compartment) is equal. This is not the case in stem cell systems where there are multiple rounds of proliferation which cause cells to amplify in number. This is how 10^5^ hematopoietic stem cells can populate a hematopoietic system with 10^9^ − 10^10^ of certain types of cells. Due to this violation of a key linear chain assumption, we instead opted to model differentiation as a simple average discrete delay.

In addition, the *α* coefficients represent the amplification of the number of cells as successive rounds of reproduction and differentiation occur when cells transit from the HSC compartment to a final mature stage. This amplification is also modulated by the lineage bias terms from Eqs 1 and 2. Lastly, there is a cell death term in expression II. This term, like the death term for stem cells, can be impacted by niche status.

### Modeling competition dynamics of healthy and mutant clones after transplantation into variable bone marrow microenvironments

For multiple types of hematopoietic malignancies, including multiple myeloma [22], acute lymphoblastic leukemia [23] and acute myeloid leukemia [24], autologous BMT remains a widely implemented therapy. However, it has long been acknowledged that stem cells can only properly grow to fulfill physiological function when placed into the proper microenvironmental niche [25]. Additionally, it is increasingly understood that the bone marrow microenvironment also degrades with age and impedes the ability for HSCs to properly function. One prominent process is the loss of HSC-supporting endothelial cells via a reduction in vasculature within the bone marrow [13]. Specifically, type H endothelial cells are responsible for production of stem cell factor (SCF) which is crucial to HSC maintenance. SCF has been implicated for roles in both homing as well as promoting HSC self-renewal and survival [26]. In addition, defects in HSC homing mean that stem cells have a more difficult time entering the bone marrow from circulation to proliferate. From a bone marrow point of view, this is a functional loss of stem cells since HSC function is very microenvironmentally controlled [27].

Further, the lower degree of vascularization has been also attributed to lower levels of nitric oxide (NO) in aged bone marrow. This can cause greater oxygenation, since low NO levels trigger vasodilation, and increased damage to HSCs via reactive oxygen species (ROS) [28]. This production is on top of the fact that aging bone marrow has been shown to exhibit greater pro-inflammatory signaling which further leads to ROS production and toxicity for HSCs. Specifically, damage associated molecular patterns (DAMPs) increase in aged bone marrow and trigger TNF*α* and IL-6 signaling, which lead to ROS release and associated apoptosis [29].

Taken together, there is compelling molecular and experimental evidence that the aging bone marrow is far more hostile to hematopoietic cell survival than young, healthy marrow due to declines in niche quality and function. However, the quantitative impact has been so far unexamined in the context of clonal competition dynamics. In order to understand how this degradation of the microenvironment into which stem cells are transplanted influences their competition and repopulation dynamics, we varied the levels of niche degradation and cell death:

$$\begin{array}{c} \delta_{H}=\delta_{H_{0}}+\Delta ,\delta_{L_{0}}+\Delta ,\delta_{M_{0}}+\Delta . \end{array}$$ (6)

The death rate of each type of cell was modeled as the sum of two components: baseline cell death ($\delta_{H_{0}}$, $\delta_{L_{0}}$, $\delta_{M_{0}}$) which would be expected in a healthy, young bone marrow and the augmented death rate attributable to microenvironmental degradation (Δ). The multiplicity of mechanisms by which the HSC niche in the bone marrow loses its integrity during aging means that Δ represents an average approximation. However, we have attempted to understand the impact that its variation could have with our sensitivity analysis (see Results).

### Model parameterization

In order to parameterize our simulations, we used values inferred from the literature and previous modeling works (Table 1).

**Table 1:** Model parameters for healthy HSC clones.

Parameter	Variable	Value	Source
$r_{H_{1}}$, $r_{H_{2}}$	HSC Growth rate	1/10 cells^−1^day^−1^ (but variable)	[30, 31]
*h*_1_, *h*_2_	Competition coefficients	1 − variable	Extrapolation based on common cytokine pool
*K*_*H*_	HSC carrying capacity	10,000 cells	[31, 32]
*γ*	Differentiation demand coefficient	0.1	This was a scaling term introduced by us to reflect demand at biological homeostatic levels
$\delta_{H_{0}}$	HSC death rate	0.001 day^−1^	[32, 33]
*ϕ*	Myeloid bias in differentiation	0.6	[34, 35]
*α*_*M*_	Myeloid amplification rate	1000	[36]
$\delta_{M_{0}}$	Myeloid cell death rate	0.25 day^−1^	[36–38]
*α*_*L*_	Lymphoid amplification rate	1000	[36]
$\delta_{L_{0}}$	Lymphoid cell death rate	0.1 day^−1^	[39, 40]
*τ*	Maturation Delay	14 days	[41, 42]
*K*_*M*_	Myeloid Carrying Capacity	90000 cells	[36]
*K*_*L*_	Lymphoid Carrying Capacity	10000 cells	[36]

### Hematopoietic growth rates and competition coefficients

Measurements of HSC growth rates in the literature are challenging due to the variable activity states of stem cells [30]. While most cells are in a quiescent state, cell cycling does occur on a long timescale but with significant differences in observed rates. Spatially, this is represented by separate niches near either the vasculature or the endosteal surface of the bone [43]. Oxygen gradients along this axis are believed to be the main determinant of stem cell activity. Greater oxygenation near the vasculature, versus the more hypoxic bone surface, means that stem cells grow at a faster rate in the vascular and perivascular niche [32]. Studies of relative rates have also been carried out to find that the replication rate of HSCs is at least 10 times larger than their apoptosis rate [44]. Importantly, the values of *r*_1_ and *r*_2_ represent the *maximum* HSC growth rates under strong demand. Previous work by Stiehl et al. have implemented a similar growth rate for modeling clonal dynamics [45]. Stem cells are given a maximum possible self-renewal rate which becomes regulated by feedback control.

For our model, we used various stem cell growth rates in order to understand how this biological variability might impact hematopoietic dynamics. A general order of magnitude approximation based on literature values was used to reflect the heterogeneity in growth rates that has been observed. Some *in vivo* estimates of HSC replication rates have suggested roughly 1 cell replication every 14 days [31]. This is representative of a general estimation of HSC growth rates being in the range of 0.1–0.01 cells/day. For our model we chose a baseline of 0.1 cells/day but evaluated growth rates smaller than that by altering the growth ratio between the two clones (*ρ*).

Stem cell growth is moderated by competition between mutant and healthy clones. This is biologically rooted in the fact that HSCs are competing for a finite pool of pro-growth cytokines [46]. In fact, it is the variation of these cytokines during exceptional biological circumstances, such as emergency hematopoiesis, which allows the body to regulate and augment stem cell activity [46]. Specific growth factors, such as erythropoetin, also elicit targeted effects for hematopoietic daughter lineages which might be in greater demand at a given time [47]. Pro-growth cytokines are also used therapeutically in the form of granulocyte colony stimulating factor (G-CSF) to help patients’ recovery from chemotherapy [48, 49]. However, in spite of the mechanistic underpinnings of the competition dynamics for cytokines, quantitative measures between stem cell clones in the bone marrow remain elusive. In order to acknowledge this gap in the literature, we specifically investigated a range of stem cell competition rates and their cross-interactions with other hematopoietic parameters.

A value of *h* = 1 was used as a basis in our analysis as a healthy baseline which we varied to understand its impact (see Results). This is reasonable because in a healthy hematopoietic system there would be no difference between mutant and healthy cells. They exert the same amount of competition intensity on each other and they are governed by the same carrying capacity. Mathematically, this is reflected by how when *h* = 1 and *r*_1_ = *r*_2_, Eqs 1 and 2 are identical.

### Stem cell carrying capacity, differentiation, and death rates

Stem cell carrying capacities, as mentioned above, are limited by available pro-growth cytokines. Literature estimates have centered on a total HSC population of 10,000 cells [32, 44]. HSCs have been recognized to be a heterogeneous group of cells with differing levels of activity. This corresponds to, among other differences, their localization in separate niches in the bone and the growth rate differences that are attendant. But even in spite of the variation in HSC repopulation kinetics after transplantation, a general carrying capacity of 10,000 cells has been observed in experimentation and also shown to be robust in reproducing patient dynamics in modeling [31, 32]).

Stem cell death and clearance rates were harder to determine due to the longevity and self-renewing abilities of HSCs [33]. Our estimates are derived from modeling studies which have tried to reproduce experimental and animal data. In these cases, stem cell apoptosis rates were significantly lower than the growth rates (1/10 of the value). However, models have found this growth rate to be sufficiently low that its variation has a relatively minor effect on outcomes [32]. This supports the experimental model view that HSCs are very long lived, and whose dynamics are not significantly impacted (at least in a healthy state) by their apoptosis rates. We chose a very small death rate that is 1/100 of the growth rate in order to emphasize this. However, the impact of increasing death rates was also investigated when exploring the cancer dynamics (see Results).

Lastly, we introduced a differentiation term, *γ*, in order to reflect the demand of asymmetric differentiation on loss from the HSC compartment. In general, stem cells characteristically have the ability to replenish daughter cell populations without leading to significant loss of their own through asymmetric division. However, symmetric (HSC-loss) division is still possible due to heightened physiological demand. Previous modeling works have examined how this loss is controlled by the larger state of the hematopoietic system [16]. We chose to mimic this approach and use a differentiation term which, at maximum, could be on the same order of magnitude as the stem cell replication rate (similarly used in [32]). However, in practice, since the model simulations were not in such extreme conditions, the demand was an order of magnitude lower than the replication rate.

### Lineage bias and maturation delays

Other important parameters for our model were the asymmetric allocation of daughter cells to myeloid vs lymphoid cells as well as the delays in maturation. Lineage commitment is an important step in stem cell differentiation during hematopoiesis, but cells do not become lymphoid or myeloid in equal proportions. We used a generally agreed upon proportion of 3:2 myeloid:lymphoid cells. Previous studies [34] found similar ratios through *in vivo* murine studies. In humans, the ratio is similar, although there is the complicating factor in that there is variation throughout life. The murine approximation of 3:2 is what we chose as a relatively balanced approximation given that human ratios change from lymphoid dominant to myeloid dominant by approximately the age of 21 [34]. This has also been supported in studies which measured common myeloid and common lymphoid progenitor cells and imbalances caused by secreted osteopontin (Supplementary figure 1 in [35]).

Maturation delays for daughter hematopoietic cells were highly variable since myeloid and lymphoid cells are heterogeneous populations. Neutrophils, for example, have a delay lag time of approximately 5 days as measured in [42]. However, more detailed RNA studies have shown differentiation to occur after as long as 14 days [41]. We chose this estimate of 14 days as a baseline to cover the breadth of phenotypic change that cells undergo during differentiation. We believe this is biologically reasonable and our investigation of delay variation showed how this maturation time could interact with disease (see Results).

### Daughter cell carrying capacities, amplification rates and death rates

Daughter cell amplification factors were based on the modeling done in [36]. We used their method of describing amplification rates of daughter cells based on a multiplicative factor of stem cell division. They have modeled this amplification factor as on the order of magnitude of a 10,000-100,000 fold change from stem cells to daughter cells. We have followed this and use a parameter of 10,000 times the HSC differentiation rate (*γ* = 0.1).

Daughter cell carrying capacities were also based on the modeling of [36]. While they had variable final capacities as a function of body mass and feedback parameters, we chose a more simplified method of fixing the total capacity at 100,000 cells. This was because we did not seek to model the entire hematopoietic system but instead a subset of two connected niches. In doing so, we used 100,000 as an estimate of the ‘local’ carrying capacity for these connected niches. Our model was intended to present a local, view of repopulation dynamics. Within these 100,000 myeloid and lymphoid cells, we further divided the ratio as 9:1 in favor of myeloid cells. While the exact composition can vary [34], we used this to represent how lymphoid cells typically exit the marrow more than myeloid cells.

Finally, hematopoietic daughter cell death rates were another highly variable parameter for which we used an average estimate. Lymphocyte death rates were used from [39], which assumed death rates are dependent on IL-2 cytokine concentration but generally in the range of 0.1 day^−1^. In terms of specific cell types, effector T cell populations have been indicated to have clearance rates of 0.05 day^−1^, which is also similar to the value we have chosen [40]. Myeloid death rates are even more variable due to the heterogeneity of mature myeloid cells. However, their turnover is characteristically higher, especially when they are recruited to fight infection [38]. Neutrophils have also been found to have a half life of hours [37]. Our value of 0.25 day^−1^ reflects their faster turnover and cell death.

### Sensitivity analysis of the impact of varying competition and growth rates

We analyzed the impact of variation in both competition strength between the clones as well as their relative growth rates. From Eqs 1 and 2, the growth rates of the healthy and mutant clones are *r*_1_ and *r*_2_, respectively. In order to simplify the parameter variation, for our analysis we set *r*_1_ as a constant (Table 1) and then set *r*_2_ as a multiple, *ρ* of *r*_1_:

$$\begin{array}{c} r_{2}=\rho r_{1},r_{1}=0.1\;{\text{cells}}^{-1}{\text{day}}^{-1} \end{array}$$ (7)

This allowed us to treat *ρ* as the relative growth rate of mutant clones as compared to healthy HSCs.

All other parameters were held constant at the values in Table 1.

In addition, initial population numbers of the cell types that were modeled were set at 100 cells each to represent a small initial population of cells from which the hematopoietic system would be reconstituted. For the delay differential equations, the historical values for times *t* < 0 were also set at 100 cells for each cell type. While this could have been changed, we believed that a constant history buffer of this size for each population would accurately represent hematopoietic populations regrowing in nearly empty niches.

### Quantifying differentiation demand at homeostasis

In Eqs 1 and 2, stem cells differentiated into both myeloid and lymphoid daughter cells. The rate of loss by differentiation from the HSC compartment was modeled as:

$$\begin{array}{c} DH_{i}\;\text{where}\;D=\gamma \left(\phi \sigma_{M}+\left(1-\phi \right)\sigma_{L}\right) \end{array}$$ (8)

This value, *D*, represents the differentiation demand the HSC compartment experiences due to feedback to satisfy myeloid and lymphoid demand. In our simulations of HSC growth as described above, we used *D* as a measure of differentiation demand and measured it at homeostasis at the end of the simulation.

### Simulation of hematopoietic growth post-transplantation: Initial conditions and parameters

Myeloablative treatment of patient bone marrow is a traditional precursor to hematopoietic stem cell transplant [50]. While other options have been used, high dose chemotherapy regimens have been widely employed in acute myeloid leukemia (AML), acute lymphocytic leukemia (ALL), chronic myelogenous leukemia (CML), and other myeloproliferative disorders due to their ability to offset the toxicity impacts of total body irradiation (TBI). While TBI has been shown to have a dose-dependent increase in the ability to prevent relapse, studies have shown that this is often offset by transplant-related deaths due to secondary radiation-related toxicities [51]. Chemotherapy-only regimens have been devised to address this toxicity by using a combination of high dose alkylating agents, busulfan and cyclophosphamide, to still achieve maximal leukemic cell death and clearance of host hematopoietic cells.

Myeloablative chemotherapy before hematopoiesis has profound differences when compared to total body irradiation. HSC visualization studies have shown that irradiation destroys sinusoidal cells, one of the anchors of the vascular niche in the bone marrow [43, 52]. In contrast, busulfan and cyclophosphamide have directed effects to the hematopoietic cells, including nonproliferating ones, and leukemic cells [50, 53].

To simulate post-transplantation hematopoietic growth dynamics after myeoablative chemotherapy, each empty niche was seeded with 100 stem cells, lymphocytes, and myeloid cells. This mimics the immediate state of homing of transplanted cell populations to the different niches. Growth was then simulated using the parameter set in Table 1 as default, except for specific parameters that were varied for investigation. The total growth time of the simulation was 280 days with a temporal resolution of 1/100 of a day. In determining the length of time to run the simulation, 280 days was found to be long enough to establish steady state with little change after this time. In simulations where aged bone marrow was studied, the degradation-augmented death rates (Eq 6) were used instead of the baseline death rates.

Lastly, we also simulated situations in which growth factor support was employed to restore proper microenvironmental support for hematopoietic cells. This rejuvenation was simulated as a removal of the degradation-augment on the hematopoietic cell death rates on day 15 of the simulation (Δ = 0 when t > 15 days). While the day of simulated growth factor impact was arbitrary, using a Heaviside function (i.e. immediate effect) to simulate the sudden removal of disease, represents scenarios where treatment can lead to dramatic and rapid improvements in patient hematopoietic health. Furthermore, another advantage of our modeling of microenvironmental impact is that a reduction in hematopoietic cell death due to treatment is not solely tied to one type of growth factor or treatment mechanism in supporting the transplanted cells. While we focus on the use of growth factors, supportive treatments for transplanted cells also help engraftment and stem cell homing. This overall improves stem cell retention in the marrow and reflects a low Δ versus damaged marrow.

### Simulation software

A numerical integration scheme (Runge-Kutta 4/5 method) was adapted for delay differential equations (DDEs) in order to solve, numerically, the system of equations for our model. Source code for the simulation is available at osf.io/f7gqb. All simulations were coded and visualized using Python (version 2.7, Python Software Foundation, www.python.org/) and the Scipy, Numpy, and Matplotlib computing libraries.

### Mouse LSK measurements

Mouse LSK measurements were taken from C56BL/6J mice purchased from The Jackson Laboratory (strain 000664, www.jax.org). Bone marrow samples were from five young mice (aged 3 months) and six old mice (aged 11 months). Bone marrow was harvested using a protocol modified from [54]. Harvested marrow was then prepared for fluorescence activated cell sorting (FACS) measurement. Harvested bone marrow were lysed with ACK lysis buffer (ebiosciences) and then were stained on ice for 30 minutes with a cocktail of antibodies; Fc Block (Becton Dickinson), biotin-conjugated anti-CD3e (clone 145-2C11, Biolegend), biotin-conjugated anti-CD4 (clone RM4-5, eBioscience), biotin-conjugated anti-CD8 (clone 53-6.7), biotin-conjugated anti-CD11b (clone M1/70, Becton Dickinson), biotin-conjugated anti-CD19 (clone 6.D5, Biolegend), biotin-conjugated anti-CD127 (clone B12-1, Becton Dickinson), biotin-conjugated anti-B220 (clone RA3-6B2, Biolegend), biotin-conjugated anti-Gr1 (clone RB6-8C5, Becton Dickinson), and biotin-conjugated anti-Ter119 (clone TER-119, Biolegend). Afterwards, harvested whole bone marrow samples were stained for one hour on ice with: streptavidin-APC-Cy7 (Becton Dickinson), APC-conjugated anti-CD117 (clone 2B8, Becton Dickinson), PE-Cy7-conjugated anti-Sca-1 (clone D7, Becton Dickinson), Pacific Blue-conjugated anti-CD48 (clone HM48-1, Biolegend), PE-conjugated anti-CD135 (clone A2F10), BrilliantViolet510-conjugated anti-CD150 (clone TC15-12F12.2, Biolegend), FITC-conjugated anti-CD34 (RAM34, eBioscience). Flow cytometry was run on a FACS LSRII and analyzed with FlowJo software (Treestar Inc.). Total LSK measurements were extrapolated based on the total cell counts and an established surface marker profile [54].

## Results

### Clonal competition reduces hematopoietic productivity and exacerbates the impact of growth rate disparities

The intensity of competition and difference in growth rates between mutant and healthy HSC clones are two significant, but poorly characterized, parameters of CHIP. Measuring competition and growth differences is challenging due to the nonlinear accumulation of mutations with age and the variable impacts of the acquired mutations [1, 3]. For example, while mouse models with mutations in both copies of p53 exhibited greater HSC growth than wild-type mice, there were no differences between wild-type and p53 mutation heterozygotes [55]. Ecologically, competition and growth rates are important for predicting whether mutant clones dominate healthy clones. Furthermore, the ultimate outcome is not obvious since there may be interactions between competition intensity and growth rate differences.

In order to clarify how these two ecological variables may impact hematopoietic trajectories after transplantation, our model first examined the impact of competition between healthy and mutant HSCs. The tested outcomes were compared to the dynamics of a healthy patient without CHIP. Specifically, a healthy patient would have no divergence between healthy and mutant cells (i.e. there are no mutant cells). In this case, the competition coefficients should be equal (*h*_1_ = *h*_2_ = 1) and there should be no growth rate disparity (*ρ* = 1). Under these conditions, the two clonal HSC populations modeled in Eqs 1 and 2 become equal.

Our analysis focused on the case where symmetric competition intensity is occurring (*h*_1_ = *h*_2_) between mutant and healthy clones. Ecologically, this represents a situation of significant overlap between the niches of healthy and mutant HSCs. In this case, the mutant and the healthy HSCs exert similar competitive pressures on each others’ populations. There is significant evidence to suggest that such a dynamic is a good approximation for CHIP and pre-malignant, pre-clinically-cancerous hematopoiesis. Spatially, HSCs are extremely dependent upon occupying the correct niche in order to sustain their populations [43]. Even when hematopoietic mutants have become clinically cancerous, these new leukemic stem cells are still dependent upon the same niches as healthy stem cells [56]. A situation where competition intensity would not be symmetric might be when the niche of the mutant clone is a superset of the niche of the healthy clone. This occurs in metastasis when leukemic cells have been liberated from their constraint to bone marrow stem cell niches and can move to other organs. Here, the competitive impact of healthy cells on mutant cells would be rather low. But the competitive effect of mutant cells on healthy cells would still be high since leukemic cells are still competing for bone marrow niches (*h*_2_ > *h*_1_). However we restricted our analysis to pre-cancerous mutant HSCs, as is the case in CHIP, and took the more limited approximation that the relative competitive impact of each clone on the other is approximately equal.

**Fig 2:**
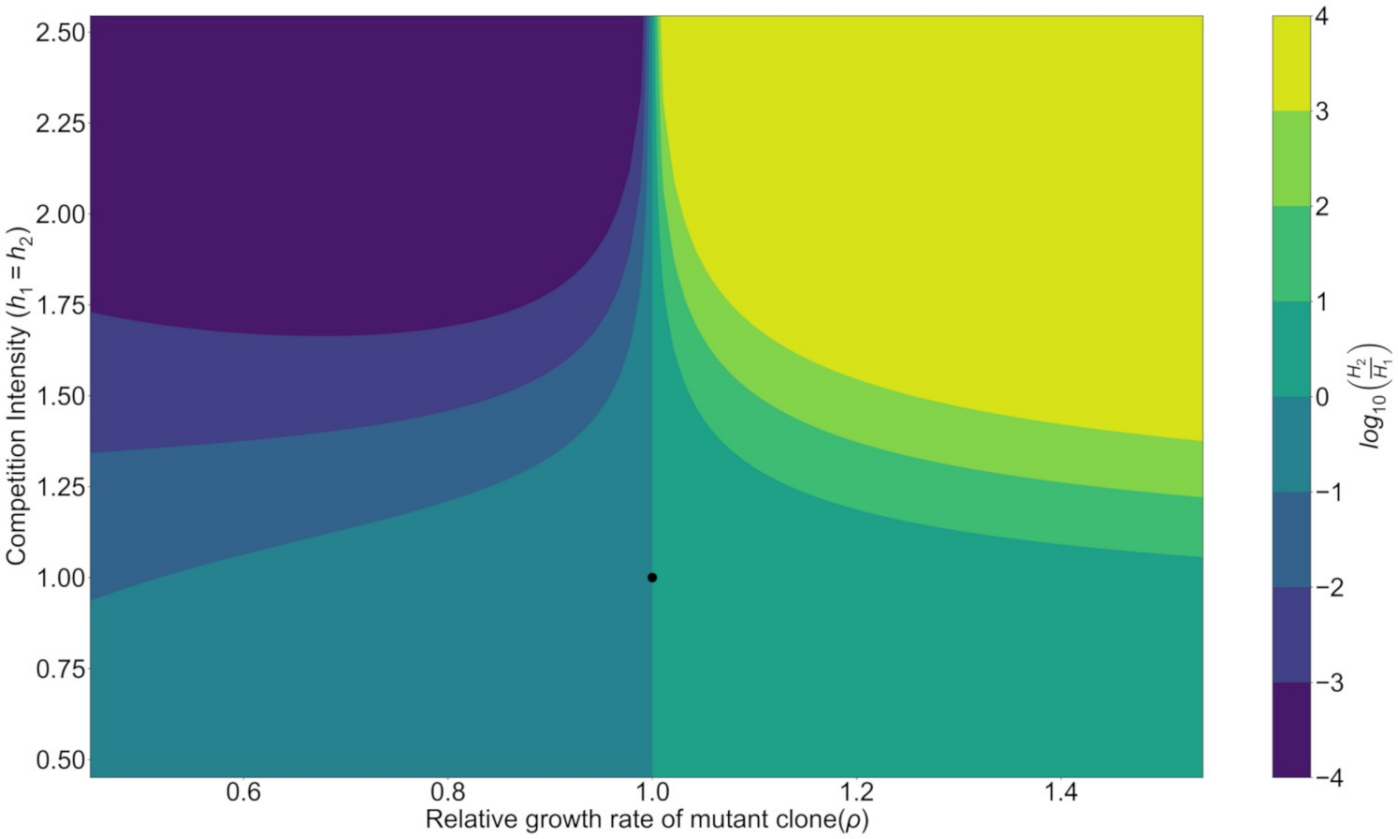
Final stem cell ratios for different growth rates and competition intensities. The growth rate discrepancy, *ρ*, exerts a dominant effect upon whether the final population sizes favor healthy HSCs over mutant HSCs. If the mutant growth rate is higher, then it will be become the more dominant clone. However, the competition intensity between the healthy and mutant clone is also important in that it exacerbates the degree of asymmetry in the final composition. The black dot represents the ecological parameters of a healthy patient.

**Fig 3:**
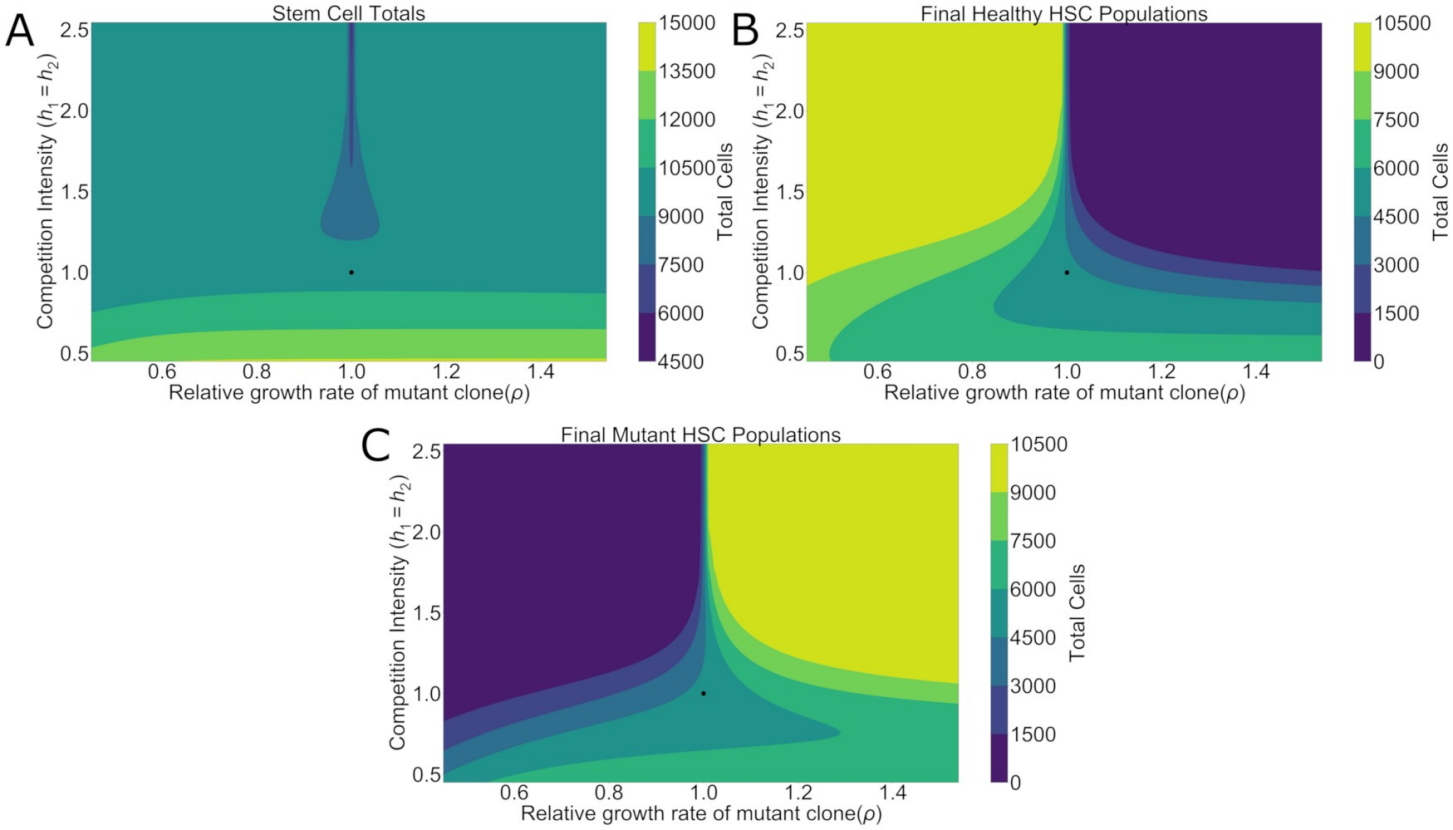
Total stem cell values as a function of growth rate difference and competition intensity. **A**. In determining the total number of stem cells, the competition intensity plays a dominating role when *h*_1_ = *h*_2_ < 1. In this region, as competition decreases, total stem cell numbers increase, allowing them to reach final population numbers above normal carrying capacity. **B**. When breaking down the actual composition of the population, healthy HSCs are competitively excluded for larger mutant growth rates and greater competition intensities. **C**. Similarly, mutant HSC populations become extinct when competition intensity is high but they have a lower relative growth rate to healthy cells. Black dots represent the parameters of a healthy patient.

Under this regime, hematopoietic repopulation was simulated after the autologous transplantation of a population of cells into the empty bone marrow. Competition intensities and relative growth rates were varied to understand their impact on both the ratio of mutant and healthy stem cells (Fig 2) as well as the final overall stem cell numbers (Fig 3). The relative ratio was examined as an indicator of clonal dominance in the hematopoietic system after repopulation. However, the total number of stem cells was also critical to understanding the downstream production and ability for the bone marrow to fulfill physiological demand.

A primary observation was that final hematopoietic compositions favored the faster growing clone, but increasing competition served to magnify this imbalance. In situations where inter-clonal competition was less intense than healthy patient levels (*h*_1_ = *h*_2_ < 1), competition had a minimal impact in determining the final clonal ratio. While there was still a skew towards the more rapidly growing clone, there was no competitive exclusion of the slower growing stem cells. Overall, when competition intensity was below normal levels there was an increase in total stem cells. While it may first seem paradoxical that these total population sizes are larger than the carrying capacity (*K*_*H*_), this is because the carrying capacity is, ecologically, determined by competition. Under Lotka-Volterra competition dynamics, if *h*_1_ = *h*_2_ = 1, then the two stem cell clones are exerting the same competition pressure upon each other. This means that, if all other parameters are equal, *H*_1_ and *H*_2_ are essentially halves of the same population of stem cells. Their final populations will be $\frac{K_{H}}{2}$ for each clone. However, if competition intensity decreases so that *h*_1_ = *h*_2_ < 1, then each clone can reach a final population value greater than before and the sum of *H*_1_ and *H*_2_ will be greater than *K*_*H*_. Biologically, total population sizes greater than the total carrying capacity simply reflect the fact that the mutant and healthy HSCs are no longer competing for the same set of resources and so their total numbers can grow to be greater than *K*_*H*_ even though each individual clone size remains smaller than *K*_*H*_.

Competitive exclusion became much more apparent when competition intensities were greater than normal levels (*h*_1_ = *h*_2_ > 1). For a given discrepancy in growth rate, as competition intensified, so too did the imbalance between healthy and mutant stem cells. In situations of extreme clonal competition, even small differences in growth rate led to competitive exclusion of the slower growing lineage. Importantly, though, even though there was dominance of one clone over the other, the total number of stem cells remained unchanged (Fig 3A). The only exception was when *ρ* = 1. Under this condition, with indicates no growth rate difference, greater competition led to final HSC populations which were lower than those of a healthy patient.

**Fig 4:**
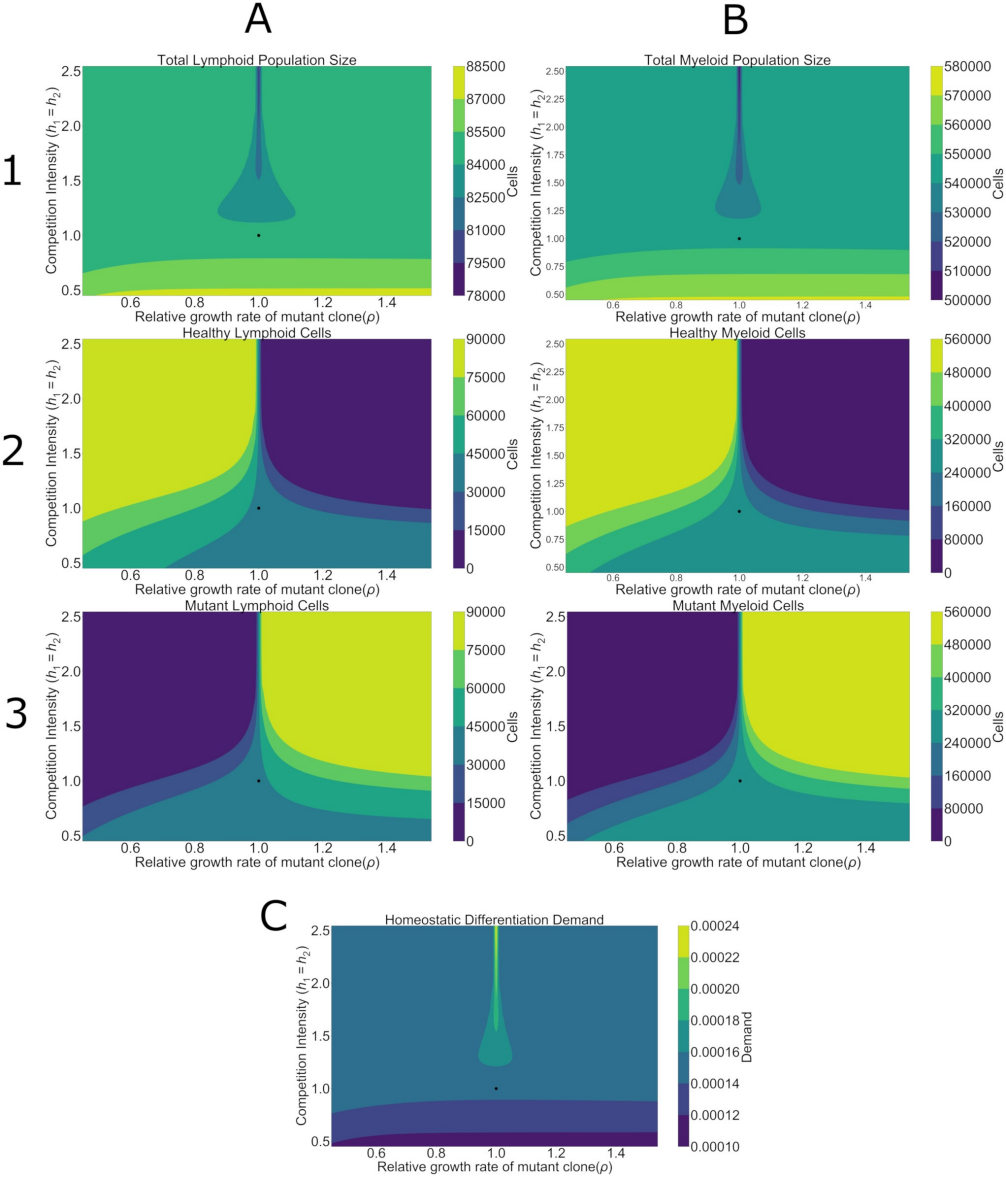
Total myeloid and lymphoid populations sizes and differentiation demand. Growth rate and competition effects are similar to the impact on HSCs. Lower competition leads to greater production of myeloid (column **A**) and lymphoid (column **B**) cells. Accordingly, there is also a relationship with demand at homeostasis (panel **C**). There is a small parameter region where the total production declines, which corresponds to an area where the relative growth rates of the two mutant clones are similar (*ρ* = 1). However, just looking at the total (row **1**) production of each cell type does not reveal the dominance of healthy (row **2**) or mutant (row **3**) cells which is dependent on growth rate disparity. Black dots represent the parameters of a healthy patient with minimally divergent healthy and mutant clones.

These upstream stem cell impacts of competition and growth rate were propagated to downstream daughter cells and physiological demand on the hematopoietic system (Fig 4). Previous experimental and theoretical studies have shown that stem cell dynamics have a significant determining effect on differentiated hematopoietic daughter cell dynamics [19, 36]. These top down effects were similarly present in our model in the context of clonal competition. Similar to stem cells, in low-competition regimes the lymphoid and myeloid daughter cells both exhibited the increased final populations relative to healthy baselines. In addition, weaker competition also allowed the coexistence of myeloid and lymphoid cells from both the healthy and mutant clones. Finally, this increased productivity was also reflected in the physiological demand at homeostasis. Greater daughter cell populations meant reduced differentiation demand on HSCs (Fig 4C).

Lastly, as with the stem cells, greater competition led to competitive exclusion of daughter cells from the slower growing lineage. This is unsurprising as exclusion of stem cells would be predicted to lead to a decrease in their daughter cells. Moreover, it emphasizes the systemic impact that competition has on exacerbating stem cell growth rate differences. Greater competition at the stem cell level will lead to a more extreme exclusion of both the slower growing lineage of stem and daughter cells from the final hematopoietic populations.

### Competition between stem cell clones worsens recovery in degraded bone marrow niches

In addition to understanding how repopulation after transplantation occurs in healthy bone marrow niches, we also investigated the impact of a degraded or aging-damaged bone marrow (Δ in Eq 6). A significant element of the difficulty in treating hematopoietic malignancies is that many of them occur in older patients who have compromised bone marrow conditions compared to young patients [9]. A number of aging-related declines in bone marrow quality have been studied, which lead directly to impeded HSC growth and function [13, 29].

**Fig 5:**
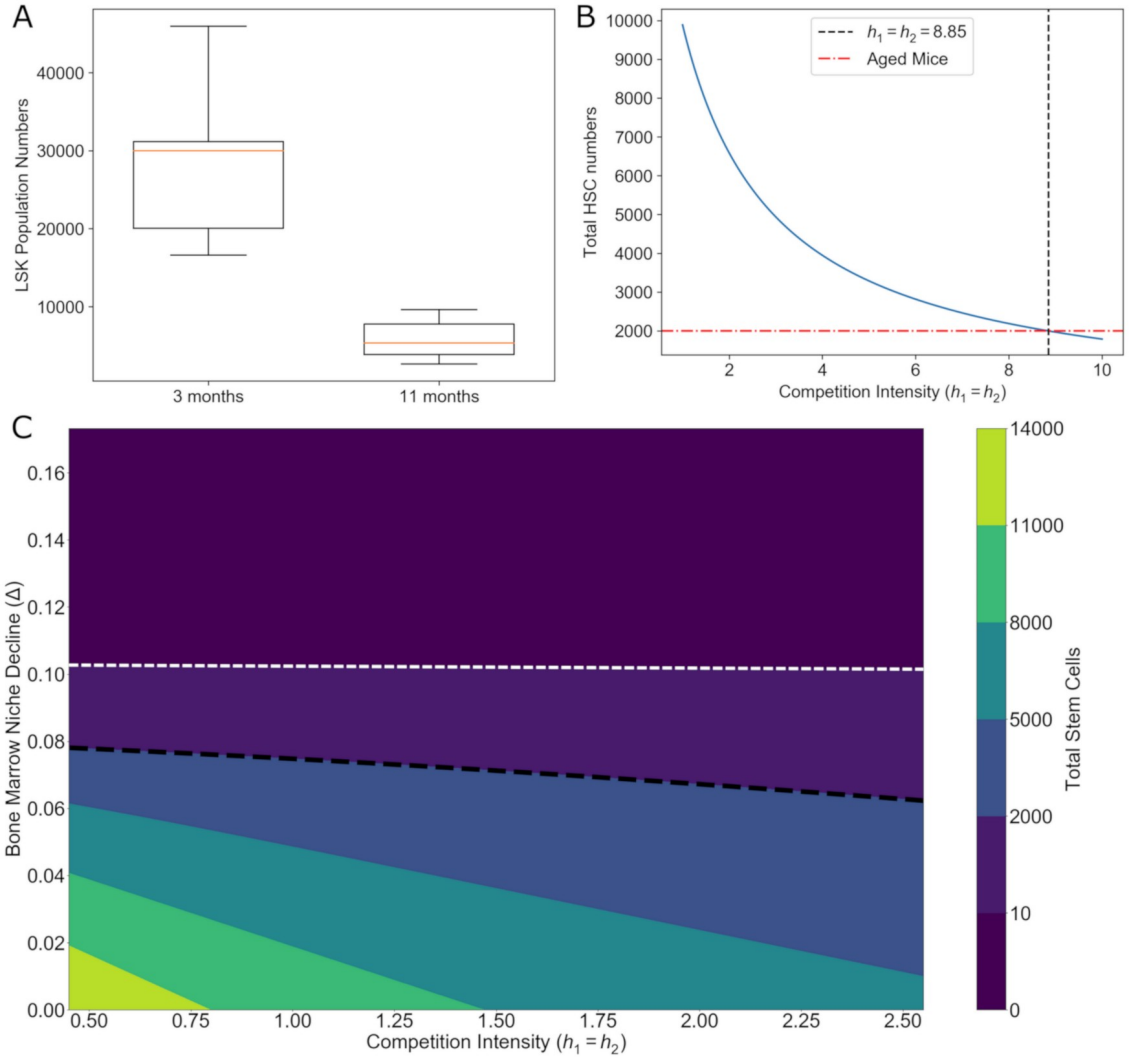
Estimation of clonal competition intensity in old mice show significantly greater inter-clonal competition but equal growth rates. **A**. LSK cell counts for a cohort for 3 month old (*n* = 5) mice were compared to 11 month old, aged mice (*n* = 6). There was an 80% decline in LSK numbers (Welch’s unequal variances t-test, *p* < 0.001). **B**. In an uncompromised bone marrow system where Δ = 0, this relative decline in HSCs to 20% of their homeostatic numbers (red line, 2000 cells) was extrapolated to a competition coefficient of *h*_1_ = *h*_2_ = 8.85 (black line). This is in contrast to an expected competition coefficient in healthy HSCs of *h*_1_ = *h*_2_ = 1. Furthermore, this could be an underestimation since more gated LSK-SLAM populations suggest true HSC declines could be much larger (see Results). **C**. Competition exacerbates the damaging impacts of bone marrow damage. Importantly, if bone marrow damage is above a certain threshold, Δ ≈ 0.1 day^−1^ (white dashed line), then the hematopoietic system is driven to extinction regardless of the degree of interclonal competition. However, for situations where the bone marrow damage is not as severe, interclonal competition increases lead to lower total HSC populations. However, when Δ is unknown, as in older mice, this relative decline can be mapped onto a combination of Δ and *h* values (black dashed line).

HSCs give rise to the committed bone marrow lineages and mature blood compartment, whereas the bone marrow niche is a vast compartment of mesenchymal or endothelial cells that produce growth factors necessary to support hematopoiesis [57]. Genotoxic stress induced by pre-transplantation regimens deplete HSCs but also damage bone marrow niche cells. Reduced-intensity stem cell transplantation or non-myeloablative transplantation is a modification of the chemotherapy or radiation dose that spare the niche compartment. The outcomes of CHIP with varying levels of damage to the bone marrow niche has not been investigated. To understand CHIP’s interactions with clonal competition in compromised niches, we measured total stem cell numbers during recovery after transplantation in variable bone marrow niche conditions (Fig 5C).

Our analysis revealed that there are two major regimes of dynamics. If bone marrow damage is too high, then that leads to a situation where, irrespective of competition intensity, the stem cell population cannot be sustained. For our analysis, the value of Δ ≈ 0.10 day^−1^ (Fig 5C, white dashed line) was the threshold above which there was stem cell extinction. This dividing boundary represents an approximate extinction threshold since simulated recoveries in niches with more than this level of damage have less than 10 HSCs at homeostasis. For values of Δ < 0.10 day^−1^, greater competition intensity led to lower final stem cell populations. Greater competition between healthy and mutant stem cell clones in increasingly degraded bone marrow niches led to lower final stem cell counts and reflected an hematopoietic system less able to meet physiological demands.

### Growth factor support may not recover healthy marrow levels of hematopoietic productivity

**Fig 6:**
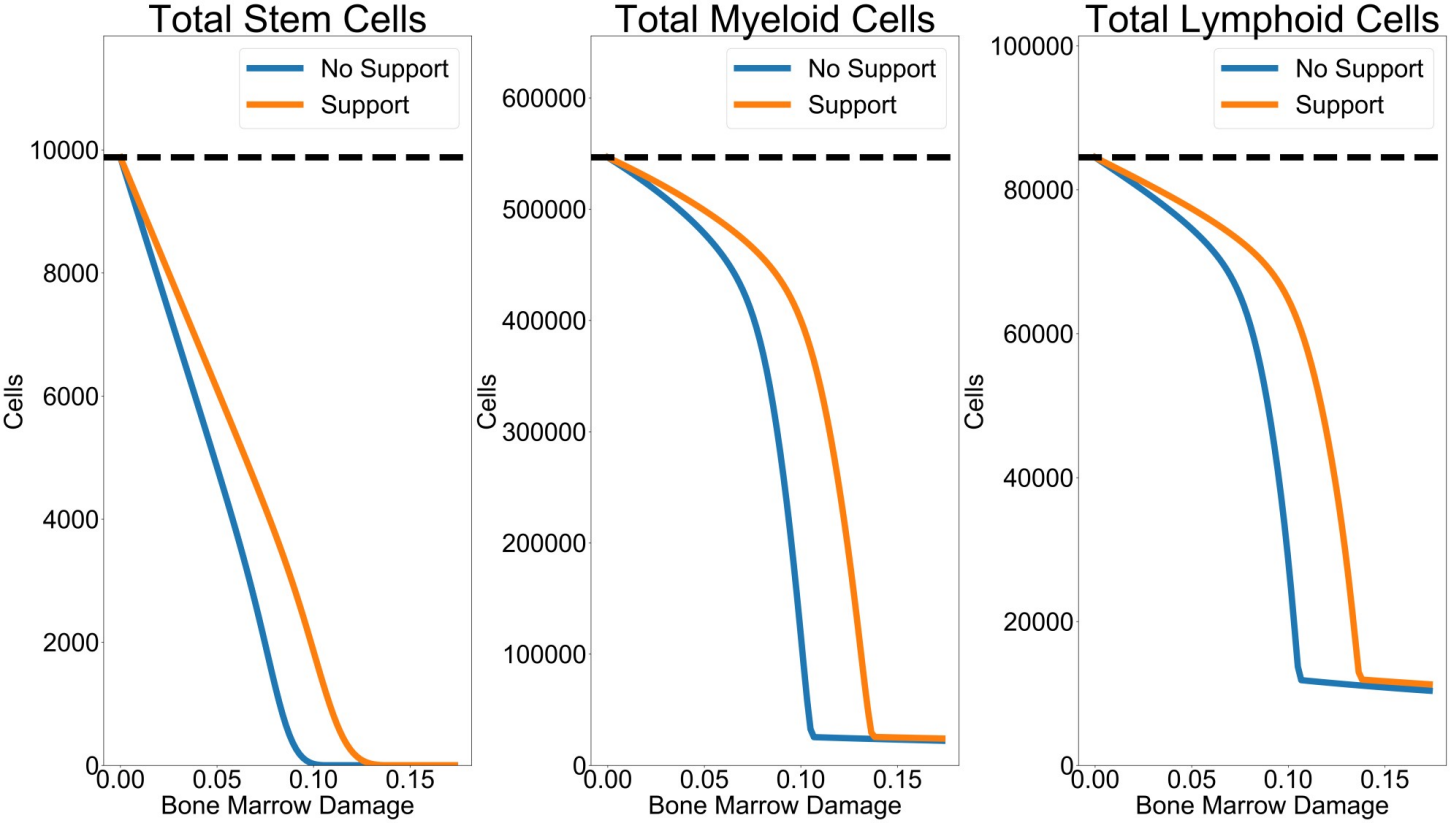
Stem cell totals after repopulation with the treatment of bone marrow condition at 15 days. Growth factor application led to modest benefits in bone marrow condition. Both HSCs, myeloid, and lymphoid cells all experienced greater final population numbers with support than without. However, growth factor support was not able to recover population numbers of a totally healthy bone marrow niche from the beginning (black dashed line). Notably, even with growth factor treatment, if the condition of the bone marrow was too poor, stem cell extinction still occurred. The flat tails of the myeloid and lymphoid cells represent residual cell populations which were going extinct but did not totally die out by the end of simulation.

In light of the impact that an inadequate bone marrow niche and clonal competition had on HSCs populations, we examined to what extent support for stem cells via growth factors could recover healthy hematopoietic productivity (Fig 6). We simulated hematopoietic repopulation after transplantation into variably-damaged bone marrow niches. However, to simulate the impact of growth factors, we then improved the bone marrow condition at 15 days after the start of repopulation (Δ = 0).

Even under the most favorable conditions, the application of growth factors to the bone marrow niche did not recover healthy hematopoietic productivity. Our simulations used healthy populations where *h*_1_ = *h*_2_ = 1 given that our previous analysis of competition intensity showed that optimal outcomes in degraded bone marrow environments occur when the competition intensity is lower (Fig 5C). However, even under this regime of minimal increased competition between clones, we still found that stem cell population numbers at homeostasis were much lower than for healthy niches. The amount of improvement from treatment increased with poorer initial bone marrow condition, but HSC extinction was still possible.

This investigation also implicitly took the effect of a maturation delay of 14 days into account. By having marrow improvement occur at 15 days, it meant that the bone marrow had remained degraded during the early post-transplantation period when there were few myeloid and lymphoid daughter cells and mostly HSCs. Stem cells had borne the brunt of the bone marrow-mediated cell death rate increases, while daughter cells had been unaffected (because they had not yet matured since the start of the simulation). By showing relatively unchanged productivity numbers between growth factor-supported and unsupported transplantation, our results indicated that it was primarily the impact of niche condition on the HSCs which led to an uncorrectable decrease in bone marrow productivity.

Based on our model, this inability to recover a healthy bone marrow system is due to the finite limits of demand on the stem cell compartment. On initial repopulation of the bone marrow, HSCs experience expansion growth as they fill niches that have been emptied before transplant. As they grow, physiological need triggers differentiation and asymmetric division from HSCs to eventually lead to mature daughter myeloid and lymphoid cells. However, a degraded bone marrow niche yields problems during this repopulation by leading to greater HSC death. This means that the overall productivity during repopulation, before a steady state is established, is lowered.

Growth factors given to support HSC populations only moderately improved bone marrow outcome because stem cells have limited productivity in terms of the maximum growth rates, *r*_1_, *r*_2_. There is a limit to the maximum flux out of the stem cell compartment, because while HSCs may have very large reproductive potentials over a patient’s lifetime, their rates of reproduction can only increase to a finite limit due to demand. Our model accounts for the fact that stem cells have great longevity (low *δ*_*H*_) but their differentiation rate is finitely limited. This reflects the fact that HSCs still have to go through the cell cycle when they need to divide—imposing biological limits on their maximum rate of differentiation. Under our model, this maximum rate of differentiation is not enough to repopulate the bone marrow when HSCs have been depleted by competition and disease and so full bone marrow rejuvenation is not possible.

### Estimation of competition intensity and bone marrow condition from empirical HSC observations in older and younger mice

Our simulated experiments highlighted the interaction between competition intensity and bone marrow condition in their ability to compromise hematopoietic productivity. However, one of the difficulties in determining the heterogeneity that may be encapsulated by CHIP is due to competitive niche interactions where multiple marrow condition/competition intensity combinations may yield the same overall stem cell numbers. Clinically, this becomes potentially problematic since gross hematopoietic numbers are often used as a marker of general health during the course of therapy [12].

In an effort to more realistically estimate what range of bone marrow declines and clonal competition intensities could lead to a given outcome, we estimated hematopoietic states from older and younger mouse bone marrow (Fig 5). Lin^−^Sca-1^+^c-Kit^+^ cells have been characterized as containing lineage uncommitted HSCs and progenitors [58] and serve as a good approximation of our modeled stem cell compartment. Marrow was harvested from young (3 month old) mice and old (11 month old) mice which displayed a significant decrease in total LSK numbers. More gated LSK-SLAM cells were also measured, but their smaller numbers and higher variability, in addition to our cohort sizes, limited our ability to detect a significant difference (see supplementary S1 Fig).

Using our model and the dynamic interactions between competition and age-related bone marrow niche decline we could then isolate a specific contour line as representing the possible bone marrow states of the older mouse cohort (Fig 5C, black line).

This range of combinations of bone marrow condition and competition intensity yields a few important insights. Firstly, the decline in total stem cell number could be explained by a broad range of competition intensities but a relatively narrow range of declines in bone marrow condition. This suggests that the 80% decline in stem cells observed in our mouse cohort is significantly influenced by the loss of niche support in the bone marrow. In terms of competition, the intensity among clones could be normal (*h*_1_ = *h*_2_ = 1) or could be significantly elevated (*h* > 2). Furthermore, our resolution in estimating the decline with age was challenged by the necessity of using LSK cells instead of the more gated LSK-SLAM populations due to statistical limitations. While we attempted to correct this by looking at the proportional decline, it is possible that there is higher variability in the LSK-SLAM population (see S1 Fig). True HSC decline could be greater than 80% which would suggest that our extrapolated competition intensity of *h* = 8.85 is an underestimation of the true degree of competition between mutant and healthy HSCs. The actual degree of competition could be much higher. This uncertainty adds urgency to efforts to have a higher-resolution study of aging niche decline and stem cell loss.

In addition, this mapping offers a way forward to estimate CHIP in terms of clonal competition intensity in patients before transplantation. Specifically, since the ability of bone marrow niches to support stem cells declines with age, this decline in bone marrow niche integrity can act as a proxy for patient age. If age-dependent niche decline could be more finely quantified, then the age of a patient and the stem cell numbers of a patient could be used to infer the inter-clonal competition intensity of HSCs (see Conclusion).

## Discussion

### Transplantation from CHIP-burdened donors can lead to the dominance of mutant hematopoietic phenotypes

Clonal hematopoiesis of indeterminate potential (CHIP) poses many challenges for predicting patient health outcomes due to the uncertain dynamics between healthy and mutant hematopoietic clones [1, 3, 59]. However one area in which the implications of CHIP have been poorly explored is how it may impact the outcomes of autologous bone marrow transplant. For a number of hematopoietic cancers, autologous stem cell transplantation remains preferred over allogenic transplantation to avoid the life-threatening potential of graft-versus-host disease (GVHD). Moreover, reduced intensity conditioning regimes have allowed transplantation in older patients due to lower stresses from chemotoxicity [50]. Transplantation in patients over 60 years of age in the US now represents nearly 40% of all autologous BMTs [10]. In these older patients, CHIP provides a complicating factor for predicting outcomes due to the possibility that sampling effects could lead to transplanted mixtures of mutant and healthy hematopoietic clones that may contribute to post-transplant leukemia.

In order to investigate the dynamics that could lead to divergent outcomes from CHIP, we employed a mathematical model of clonal competition between healthy and mutant HSCs and studied the outcomes of simulated autologous stem cell transplant.

Our model analyses addressed the open question of how variation in cell competition and growth parameters of the transplanted stem cell population could alter the final composition of marrow due to competition between healthy and mutant HSC clones. We focused on the interaction between variation in competition intensity and the variation in growth rate discrepancy between healthy and mutant clones.

Our results suggest that competition intensity between clones exacerbates the impact of growth rate differences. For divergent growth rates between healthy and mutant HSCs, competitive exclusion is not guaranteed. Instead, in order for competitive exclusion to occur, there must be greater competition intensity between healthy and mutant HSCs than would be expected in a healthy patient. This increase in competition helps to drive the slower-growing clone to extinction. In cases where mutant stem cells have a faster growth rate than healthy cells, this could lead to dominance by the mutant clone. Conversely, if the mutant is less fit than the healthy cells, it could be driven to extinction.

Studies using empirical observations of leukemic cells support our model and current conclusions. In addition to HSCs, transplantation outcomes are impacted by the stem cell niche. The degree of vascularity, oxygen availability, and stroma-derived cytokine concentration are non-uniform in the bone marrow [60]. HSCs are primarily located in the inner surface of the bone (endosteum) in specific bone marrow niches which can uniquely support HSC populations. In competitive transplantation experiments between leukemic and healthy cells, these niches have been shown to be completely dominated by either healthy or leukemic cells [56]. This represents a local, microenvironmental version of our model’s outcome and would be supported by the fact that leukemic cells are considered to have faster growth rates than healthy cells [56].

In addition, there have been notable cases in allogenic BMT which have demonstrated the ability for transplanted pre-leukemic clones to later mutate into fully leukemic populations. For example, the use of trisomy 11 as a genetic marker present in the transplantation sample was used to show the origins of leukemia 14 years after the original transplant [61]. In another situation that also involved allogenic BMT, investigators specifically used CHIP as a motivating hypothesis to identify two cases of donor cell leukemia from a population of donors older than 61 years of age [62]. While these cases are specific to allogenic transplantation, the mechanism that we have provided in our modeling framework offers an ecological explanation and understanding in both allogenic and autologous transplant settings about how CHIP and donor cell mutants may lead to leukemic clones in the transplant recipient. Moreover, our data suggest that younger donors lacking CHIP may improve the outcomes of allogenic transplantation.

A possible solution to preventing CHIP in allogenic transplants is through cord blood transplantation, which is already being implemented in the clinic. Since cord blood is collected at birth, it represents the most diverse populations of newborn stem cells without any age-related induction in clonal hematopoiesis. Thus, cord blood transplants pose a minimal risk of clonal heterogenenity with the possibility of a final, dominant mutant clone. Retrospective studies looking at cord blood transplants have shown that they can perform well enough to minimize the difference in survival in patients with and without minimal residual disease at the time of transplantation [63]. This result is notable because it is a direct test of the clonal competitiveness of transplanted cord blood populations against a known leukemic population (minimal residual disease). But even aside from cord blood, our results provide evidence for the importance in choosing younger donors who are less likely to have mutant hematopoietic clones to be transplanted both in allogenic transplantation and autologous BMT.

In the larger context of BMT, our results emphasize the risk that CHIP poses for transplant recipients. Autologous BMT, when conducted in older CHIP patients, raises the possibility of transplanting a clonal population which could lead to dominance of a mutant hematopoietic clone. Dominance of a mutant clone could then easily set up the marrow for relapse through further mutation accumulation and the emergence of a novel, cancerous population.

### Growth factor support may not be able to recover healthy hematopoietic states after repopulation

BMT and repopulation dynamics are complicated by the fact that aging leads to decline in the ability for bone marrow niches to support stem cells. Our analyses revealed that, similar to growth rate differences, increased competition between stem cell clones leads to a worsening of the already poorer outcomes due to this bone marrow niche decline. Greater interclonal competition between healthy and mutant hematopoietic cells means that the already depleting bone marrow niche is able to sustain even fewer overall cells. This leads to detrimental impacts on the fully differentiated myeloid and lymphoid cells and to a final hematopoietic system which is far from a healthy state.

A question that immediately follows from this conclusion is whether or not the use of supportive growth factors, as commonly employed in bone marrow transplants, offer benefits [64]. Clinically, there are a number of growth factor support regimens which have attempted to improve patient bone marrow condition after transplantation. By simulating the impact of growth factor support on repopulation under differing bone marrow damage conditions, we examined whether or not these treatments could improve repopulation dynamics. While gains could be achieved, the combination of maturation delay, as well as competition between stem cell clones, led to the inabilty to fully recover a healthy hematopoietic state.

Specifically, we found that while aggressive and totally restorative growth factor support could help increase stem cell numbers and total hematopoietic production, the early destabilization due to a damaged bone marrow had a lasting effect. Total production is dependent on rapid stem cell growth to large populations. This occurs as delays in hematopoietic maturation led to a lag time between stem cell population increases and daughter cell population increases. A rapid increase in stem cell numbers means that daughter cell production will approach physiologically-sufficient levels quickly. This can be considered a window in which HSCs are not experiencing negative feedback in terms of renewal and differentiation, in spite of the fact that they are actively proliferating.

However, there is a trade-off in HSC growth during repopulation in that stem cells lost due to symmetric differentiation directly take away from helping to reconstitute the HSC population. Since there is a maturation delay, even though HSCs may have reached a level that would be sufficient to fulfill physiological demand, they will still experience a large symmetric differentiation demand because daughter cell populations have not ‘caught up’. In a damaged bone marrow, this lag will take even longer because the augmented cell death and loss will functionally add more time to a response between HSC growth and daughter cell growth. This damage—even if transient—to repopulation dynamics may mean that the total cumulative production of hematopoietic cells is not sufficient to satisfy physiological demand.

This situation is made even worse due to higher interclonal competition. Once negative feedback starts to take effect as myeloid and lymphoid daughter cells are produced, it leads to a decrease in stem cell activity. However, competitive inhibition between the clones reduces the full expansion that can take place in this window of maturation delay where feedback inhibition has not yet started to increase.

In the clinic, there has been evidence to support the important role that improving bone marrow condition has in allowing hematopoietic recovery. Recently, it has been reported that after the failure of an initial stem cell transplantation, a secondary allogenic transplant was successful when mesenchymal stromal cells (MSCs) were contemporaneously intraosseously administered [57]. MSCs are responsible for creating and maintaining the stem cell niches in the bone marrow which are necessary for proper HSC function. Increasing the number of intraosseous MSCs at the time of transplantation was hypothesized to have given the microenvironmental support necessary for proper hematopoietic growth. This is a similar conclusion to our modeling results which showed that any kind of damage to the bone marrow microenvironment should be repaired as early as possible if proper recovery is to be achieved.

### Diminished stem cell populations are attributable to the synergistic impact of CHIP and patient bone marrow decline

It could be argued that while hematopoietic clonal competition dynamics are theoretically interesting, they are clinically less useful. More generally, one of the chief criticisms of the utility of CHIP in the clinic is that assays for clonal diversity or the ecological state of a patient’s bone marrow are not currently widely employed. In fact, clinical assessments of a patient’s hematopoietic state are generally limited to assessing gross hematopoietic cell population sizes [12]. It has been argued that in light of this lack of ecological (population-level and microenvironmental) assaying, and since clonal dynamics are not explicitly tested, better understanding of CHIP and patient bone marrow microenvironment can provide little utility to clinical treatment.

However, our model offers insights into the importance of measuring both the ecological condition of patient marrow as well as the clonal dynamics in transplanted stem cell populations. To highlight this importance, we compared LSK cell numbers in a cohort of old and young mice. We measured a significant (80%) reduction in LSK cells in aged mice. Based on our model, this empirical reduction in stem cell numbers was used to estimate a range of bone marrow decline/competition intensity combinations which could yield these results. Importantly, this suggests that the same dramatic loss in stem cells can be attributable to either very little CHIP but significant bone marrow decline, or vice versa. Furthermore, in terms of predictive utility, our results highlight the importance of understanding the bone marrow microenvironmental condition into which stem cells will be transplanted. Even small amounts of clonal competition could lead to the observed dramatic declines in stem cells if the bone marrow condition is sufficiently poor. Successful clinical management requires understanding CHIP in the context of the patient microenvironment.

### Limitations and future work

Our model is an initial attempt to gain a more robust understanding of how ecological competition parameters could influence final bone marrow states. However, as its focus was on the broad ecological dynamics, there are notable limitations which also provide avenues for future extension and research. One simplifying feature of the model design was the fact that the model divided HSC clones into two broad ‘healthy’ and ‘mutant’ categories. While it is clear that there are multiple types of clones present in CHIP, the exact dynamics during their origin are poorly understood. This mirrors the origins of clonal diversity in different cancers which suggest that stochasticity may have a large role in determining the dominant populations [65].

Nonetheless, while our model’s division into just two major clones is a more simple approximation, there are still insights to be gained, especially given the sensitivity analyses which can suggest how broad changes in the average mutant phenotype could impact hematopoietic outcomes. We recognize that there are alternative ways that this diversity could be modeled, including in previously published stochastic schemes [15]. Other works have sought to explore the specific impacts of mutations in leading to clonal dominance [66]. By implementing a stochastic Moran process, there is evidence that mutations in dominant clones may have arisen through stochastic clonal expansion instead of through an explicit fitness benefit. In addition, our model does not attempt to model the stochastic processes of engraftment of transplanted cells. Simulations with experimental data have shown that transplantation can be a severe population bottleneck which influences the dynamics of which clones actually engraft at the start of repopulation [67]. Our model could be extended to combine its predictions of mean-field dynamics in addition to the stochastic drift processes that are likely present in pre-leukemic stem cells. This extension could potentially help explain why CHIP may not always lead to malignancy.

Lastly, our estimate of competition parameters could be narrowed even further through research to quantify, more rigorously, the degradative impact of aging on the bone marrow. One of our major conclusions is that predicting the outcome of CHIP requires also understanding host marrow condition since multiple combinations of marrow decline and competition intensity could lead to similarly dramatic losses of stem cells. This does, however, pose a limitation in our parameter estimation, as HSC numbers could not be mapped 1:1 with the level of interclonal competition. A better understanding of the degradation due to aging would help narrow down the range of competition values by narrowing down the range of reasonably expected bone marrow damage in older patients. Current biological understanding supports a model of the bone marrow which degrades over a patient’s lifetime due to inflammation, stromal changes, and oxidative damage [29]. In fact, stem cell niche degradation due to aging is a process that has been observed to be present in tissues beyond the hematopoietic system [68].

The exact quantitative relationship between age and the increase in stem and daughter cell loss is poorly characterized. Small cohort studies of patients between 16-28 years and 63-92 years have shown increases in nitrogen oxide production and lowered Ki67 and p53 expression [69]. There are also notable declines in function of mesenchymal stem cells which are a crucial progenitor of stromal cells necesary for HSC function [70]. Extrapolating the stem cell and daughter cell death rates are more difficult, however, and are a logical avenue to extend investigation in order to help improve the utility of our modeling framework.

In conclusion, our research suggests that CHIP in the context of bone marrow transplants and repopulation dynamics should be investigated through an ecological lens. Repopulation should not be considered as a clonally homogeneous process, but instead a competitive one. Depending on the competition level, growth rate difference, and bone marrow condition, transplantation can lead to very different hematopoietic outcomes in terms of clonal composition. Clonal dominance of mutant clones may lay the seed for reemergence of disease or other hematopoietic and health disorders due to CHIP presence in donors. We advocate a further adoption of this ecological paradigm as a way of both investigating CHIP during transplantation and treating disease more effectively overall.

## Supporting information

S1 Fig LSK-SLAM populations in our cohort of old and young mice. LSK-SLAM cells displayed significantly more variability than LSK cells in a cohort of 3 month old (*n* = 5) and 11 month old (*n* = 6) mice. The difference is not significant at *p* = 0.12 based on a 2-tailed T test. The sample sizes of our cohorts here is the primary limitation in preventing us from having sufficient power to detect the differences for LSK gated cells which are in much smaller numbers than the larger LSK population.
(TIF)

S1 Model Analysis Analysis of stem cell values at stability. A key question we examined in our model was to determine the steady-state relationship between the healthy and mutant hematopoietic stem cell populations, *H*_1_ and *H*_2_, respectively. Our analysis found an expression for the homeostatic ratio of stem cell populations dependent on ecological parameters.
(PDF)
