# Supplementary figures and images for: Clonal hematopoiesis of indeterminate potential and its impact on patient trajectories after stem cell transplantation
Source: PLoS Comput Biol. 2019 Apr 26;15(4):e1006913. doi: 10.1371/journal.pcbi.1006913 (PMC6505959; doi:10.1371/journal.pcbi.1006913)

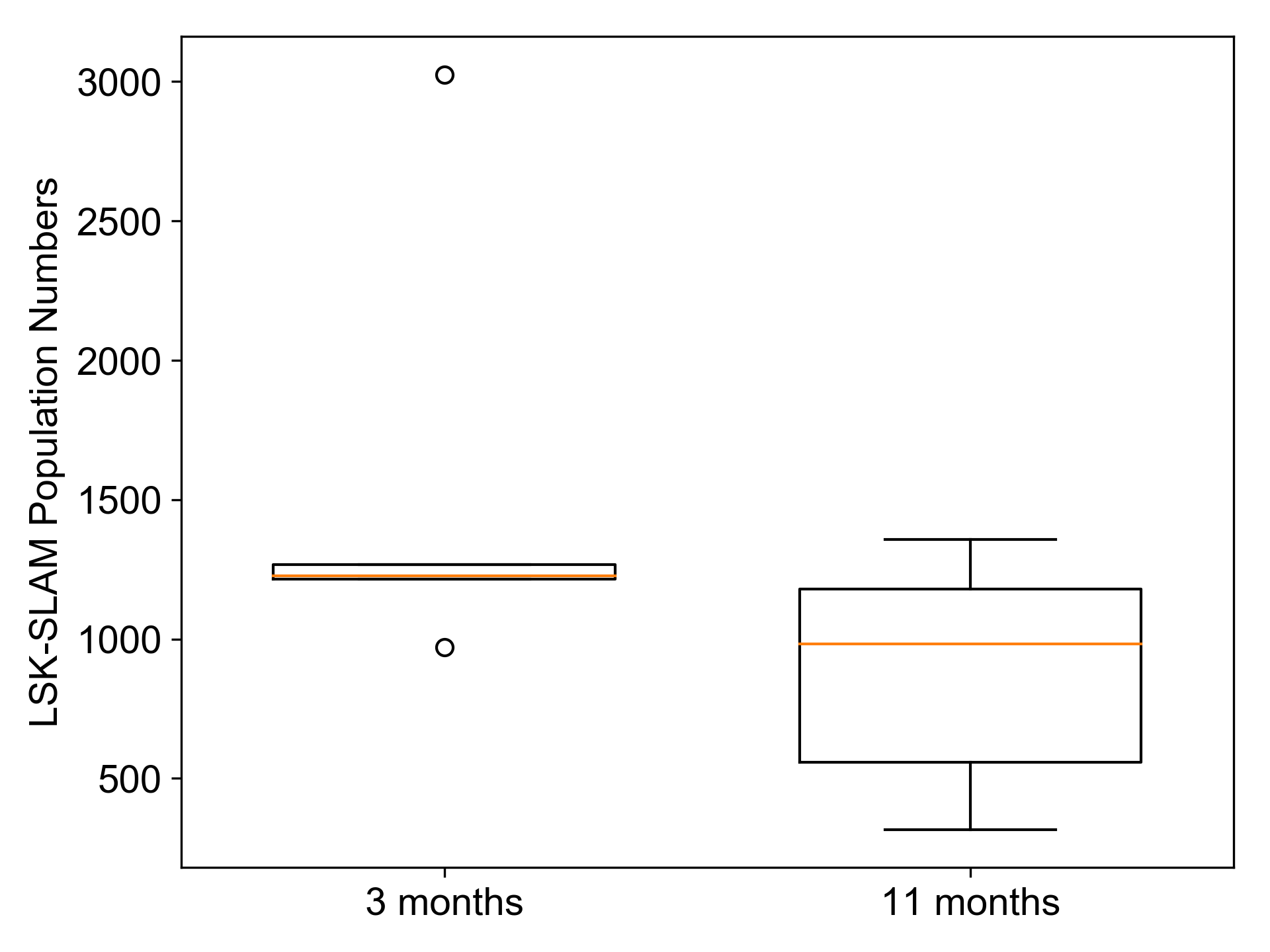

Supplement: S1 Fig — LSK-SLAM cells displayed significantly more variability than LSK cells in a cohort of 3 month old (n = 5) and 11 month old (n = 6) mice. The difference is not significant at p = 0.12 based on a 2-tailed T test. The sample sizes of our cohorts here is the primary limitation in preventing us from having sufficient power to detect the differences for LSK gated cells which are in much smaller numbers than the larger LSK population. (TIF) [file pcbi.1006913.s001.tif]
