# Supplementary material for: Clonal hematopoiesis of indeterminate potential and its impact on patient trajectories after stem cell transplantation
Source: PLoS Comput Biol. 2019 Apr 26;15(4):e1006913. doi: 10.1371/journal.pcbi.1006913 (PMC6505959; doi:10.1371/journal.pcbi.1006913)
Supplement: S1 Model Analysis — A key question we examined in our model was to determine the steady-state relationship between the healthy and mutant hematopoietic stem cell populations, H1 and H2, respectively. Our analysis found an expression for the homeostatic ratio of stem cell populations dependent on ecological parameters. (PDF) [file pcbi.1006913.s002.pdf]

Supplementary analysis for 'Clonal hematopoiesis of indeterminate potential and its impact on patient trajectories after stem cell transplantation'

Derek S. Park, Afua A. Akuffo, David E. Muench, H. Leighton Grimes, Pearlie K. Epling-Burnette, Philip K. Maini, Alexander R. A. Anderson, Michael B. Bonsall

S1 Model analysis A key question we examined in our model was to determine the steady-state relationship between the healthy and mutant hematopoietic stem cell populations, H_1 and H_2, respectively. The equations for the dynamics of the two hematopoietic stem cell clones are:

$$\frac{dH_1}{dt} = r_1 H_1 \Big(1 - \frac{H_1 + h_2 H_2}{K_H}\Big) - \gamma H_1 \phi \sigma_M - \gamma H_1 (1-\phi)\sigma_L - \delta_H H_1 \quad (1)$$

$$\frac{dH_2}{dt} = r_2 H_2 \Big(1 - \frac{H_2 + h_1 H_1}{K_H}\Big) - \gamma H_2 \phi \sigma_M - \gamma H_2 (1-\phi)\sigma_L - \delta_H H_2 \quad (2)$$

Importantly, the terms for the feedback functions, σ are:

$$\sigma_M = \exp\Big(-\frac{M_1 + M_2}{\kappa_M}\Big), \sigma_L = \exp\Big(-\frac{L_1 + L_2}{\kappa_L}\Big)$$

Our objective is to <u>solve for a ratio of H_1:H_2.</u> We start with, at steady state:

$$H_1 = 0 \ or \ \frac{dH_1}{dt} = 0 = r_1 \Big(1 - \frac{H_1 + h_2 H_2}{K_H}\Big) - \gamma \phi \sigma_M - \gamma (1-\phi)\sigma_L - \delta_H \quad (3)$$

$$H_2 = 0 \ or \ \frac{dH_2}{dt} = 0 = r_2 \Big(1 - \frac{H_2 + h_1 H_1}{K_H}\Big) - \gamma \phi \sigma_M - \gamma (1-\phi)\sigma_L - \delta_H \quad (4)$$

Which implies:

$$r_2 \Big(1 - \frac{H_2 + h_1 H_1}{K_H}\Big) = r_1 \Big(1 - \frac{H_1 + h_2 H_2}{K_H}\Big) \quad (5)$$

$$K_H - H_1 - h_2 H_2 = \rho(K_H - H_2 - h_1 H_1) \ where \ \rho = r_2/r_1 \quad (6)$$

$$(1-\rho)K_H = H_1(1 - \rho h_1) + H_2(\psi h_1 - \rho) \ where \ \psi = h_2/h_1 \quad (7)$$

$$H_2 = \alpha H_1 + \beta \text{ where } \alpha = \frac{\rho h_1 - 1}{\psi h_1 - \rho}, \ \beta = \frac{(1-\rho)K_H}{\psi h_1 - \rho} \quad (8)$$

This illustrates a linear relationship between H_1 and H_2 at equilibrium given our environmental parameters. This leads to a few key insights. First, since these equations are for real populations, we can stipulate that $H_1 > 0$. Then, the sign of the constant α determines whether H_1 and H_2 are competitive or synergistic, it establishes the environmentally-dependent parameters that can either support coexistence and dominance.

Specifically, ρ, the ratio between the two growth rates, and ψ the ratio between the influence of each stem cell upon the other clone, are the primary drivers. Furthermore, we can make a few restrictions on the interaction parameters and examine the impact on the dynamics.

An interesting condition is whether this model is supported in situations where the mutant clone has progressed to a clinically leukemic state and even been able to expand into other niches. In such a situation, since the mutant HSC population has a much broader niche than the healthy cells, we can say that $h_1 \approx 0$. H_1 exerts very little influence in the niche of H_2:

$$\alpha = \frac{-1}{-\rho} = \frac{r_2}{r_1} > 1 \text{ (since stem cells have positive growth rates)} \tag{9}$$

This is as one would expect. If the two populations of stem cells are asymmetrically interactive and the mutant clone is liberated from the traditional stem cell niche, then the carrying capacity, K_H is the only dominating term that will inhibit their growths.
